# Joint-Gated Continual Learning for Roadheader-Mounted Millimeter-Wave Radar Range Prediction in a Coal Mine

**DOI:** 10.3390/s26217051

**Published:** 2026-11-07

**Authors:** Xinzhi Li, Jianhong Zhao, Pengfei Xu, Jing Zhao, Pengxu Chen, Xinhua Feng

**Affiliations:** 1 School of Emergency Technology and Command, University of Emergency Management, Beijing 101601, China; lixinzhi@pku.edu.cn (X.L.); 25085705006xpf@ncist.edu.cn (P.X.); 2 Institute of Remote Sensing and Geographic Information System, Peking University, Beijing 100871, China; 3 Chengdu Aerospace Communication Equipment Co., Ltd., Chengdu 610052, China; cdht_z719@163.com (J.Z.); 202312012336@std.uestc.edu.cn (P.C.); fxhxinhua@126.com (X.F.); 4 School of Information and Communication Engineering, University of Electronic Science and Technology of China, Chengdu 611731, China

**Keywords:** millimeter-wave radar, mine excavation, dynamic disturbances, continual learning, joint gating, range prediction

## Abstract

Coal-mine excavation generates dust and water mist that impair optical sensing; millimeter-wave radar supports surrounding-range perception for assisted autonomous guidance, while changing conditions can cause predictor mismatch and unfavorable online updates. This paper proposes a joint-gated continual learning framework that integrates temporal-evolution context with observation–prediction consistency for selective adaptation. The temporal context characterizes local measurement dynamics and determines a state-conditioned residual tolerance; together with prediction innovation, it regulates parameter updates and online-memory insertion. Observation-stream evolution is decoupled from model adaptation, so measurements rejected for learning remain available to subsequent prediction, preserving information continuity while suppressing unstable updates. A lightweight regression predictor with bounded memory enables continual operation under limited resources. Experiments on field radar data and controlled disturbances show 1.35% lower MAE than a fixed predictor and 2.55% lower RMSE than independently source-tuned consistency-only adaptation, with a 0.09 mm MAE increase and updates decreasing from 94.83% to 85.51%. At matched learning rates, RMSE decreases by 0.63 mm versus consistency-only and 0.28 mm versus approximately rate-matched random thinning. RMSE was 206.76 mm versus 204.63 mm for Fixed, while the frozen GRU and Transformer achieved lower MAE and RMSE. At k=8, 99.11% of injected learning values are rejected. The framework supports efficient continuous sidewall-range prediction and model maintenance for assisted autonomous roadheader guidance in dust- and mist-affected excavation environments.

## 1. Introduction

Continuous measurement in complex confined industrial environments requires sensing systems to remain reliable under long-term operation and evolving operating conditions. Millimeter-wave radar provides compact, non-contact measurements for industrial sensing [1], including non-contact speed monitoring of rotating electrical machinery [2] and millimeter-wave radar measurements of small-scale rotors [3]. Reference-assisted mine-radar studies further illustrate that confined geometry, complex reflections, and changing operating conditions can introduce substantial temporal variability into underground measurements [4]. During coal-mine excavation, dense dust and water mist generated by roadheader cutting operations can degrade optical ranging, whereas millimeter-wave radar offers a complementary non-contact sensing modality with lower dependence on optical visibility [5]. For assisted autonomous roadheader guidance, multidirectional radar deployment can provide distance measurements toward the excavation face, the left and right roadway sidewalls, and rearward surroundings, supporting navigation, position perception, and clearance monitoring. Even after calibration and deployment, the statistical relationship between recent measurements and subsequent distances may continue to evolve under changing operating conditions and dynamic disturbances, gradually causing predictors fitted to source-domain data to mismatch the current process. Meanwhile, indiscriminately using every new observation for online learning can accumulate unsuitable parameter changes and affect subsequent predictions. The key challenge is therefore not only how to adapt a measurement predictor, but how to selectively regulate adaptation while continuously exploiting the available measurement stream.

Existing studies address temporal distribution shift and online adaptation from several perspectives. Concept-drift methods model evolving data relationships [6], while related adaptive strategies have been investigated for online sensor-drift compensation [7] and machinery anomaly detection under continuously changing operating conditions [8]. Continual learning further considers the balance among adaptation, retained knowledge, memory, and computational resources [9,10]. For time-series prediction, online ensembling [11], extrapolation and adjustment [12], proactive adaptation [13], and test-time updating [14,15] improve responsiveness to changing distributions. Related ideas have also been investigated for continual learning in radar semantic perception [16], changing machinery conditions [17], and task-agnostic online learning [18]. Confidence-based collaborative learning [19] and adaptation-label correction [20] further address unreliable adaptation. Classical innovation gating and adaptive filtering also regulate measurement updates through prediction residuals and noise statistics [21,22]. However, residual-based admission alone does not explicitly assess the temporal support provided by preceding measurements, while simply reducing update frequency does not ensure that admitted observations are suitable for model adaptation. These considerations motivate a joint admission mechanism that combines pre-observation temporal support with post-observation prediction consistency while preserving measurement continuity.

To address this problem, we propose joint-gated continual learning for radar (JGCL-Radar), a framework for radar measurement prediction under dynamic disturbances. The framework characterizes temporal-evolution context from recent measurement dynamics, event timing, and observation validity and uses this context to construct a state-conditioned residual tolerance. After the current measurement is revealed, its innovation relative to the archived prediction is jointly evaluated with temporal-context support to determine learning admission. JGCL-Radar further decouples observation-stream evolution from parameter adaptation: measurements not admitted for learning remain available to subsequent temporal modeling, while parameter updating and online-memory insertion are selectively regulated. Fixed source anchors and a bounded online reservoir provide finite-memory continual adaptation, and the entire framework follows a strictly causal predict–observe–update sequence. The prediction target is the next radar-reported range, and the archived pre-observation prediction provides the reference for evaluating prediction consistency and regulating subsequent online updates.

The main contributions of this study are summarized as follows:

- A joint-gated continual learning mechanism is developed for continuous radar measurement prediction under dynamic disturbances. Temporal-evolution context and observation–prediction consistency are integrated into update admission, enabling the model to selectively absorb new information according to the evolving measurement state rather than treating all valid observations as equally informative.
- A continual prediction framework is established by decoupling observation-stream evolution from parameter adaptation. Measurements not admitted for learning remain available to subsequent temporal modeling, while the gate jointly regulates parameter updating and online-memory insertion. Fixed source anchors and bounded online memory further support continual model maintenance under limited resources, with the contribution of replay explicitly evaluated rather than assumed.
- A systematic evaluation framework is established using calibrated field radar measurements and controlled dynamic disturbances. Development-only model selection, source-budget-matched controls, mechanism ablations, source-retention analysis, update-channel perturbations, sustained observation changes, and tail-error attribution are used to separate the effects of temporal-state selection, update frequency, replay, and accumulated adaptation on continual prediction performance.

## 2. Problem Formulation

### 2.1. Causal Continual Measurement Prediction

Let *t* index chronological measurement events, $T_{t}$ denote the event timestamp, $r_{t}$ the recorded scalar distance, and $v_{t}$ its validity indicator. Before the current observation is revealed, the predictor has access only to the preceding *L* events,

(1) $$H_{t}=\left\{\left(r_{j},T_{j},v_{j}\right):j=t-L,\ldots ,t-1\right\},\;T_{j}<T_{t}.$$

A feature mapping $x_{t}=\Phi \left(H_{t},T_{t}\right)$ produces the pre-observation prediction,

(2) $${\hat{y}}_{t}=f\left(x_{t};\theta_{t-1}\right),$$

where $\theta_{t-1}$ denotes the predictor parameters after processing measurement event t−1. Based on historical observations and event-time information, the one-step-ahead prediction ${\hat{y}}_{t}$ is generated before the current radar measurement $y_{t}=r_{t}$ becomes available. This prior prediction serves as the reference for subsequent prediction-error calculation and learning-admission assessment. Here, $y_{t}$ denotes the range reported by the radar calibrated before deployment, serving as the target for model prediction and performance evaluation rather than an independently measured geometric ground truth.

After observation, an eligibility indicator determines whether the event can be scored and considered for learning,

(3) $$V_{t}=1\left\{v_{t}=1,\;y_{t}>0,\;y_{t}\;\mathrm{is}\;\mathrm{finite},\;h_{t}\geq h_{\min}\right\},\;h_{t}=\sum_{j=t-L}^{t-1}v_{j}.$$

Here, $V_{t}$ indicates whether the observation satisfies the predefined record-status, numerical-validity, and historical-availability criteria for prediction evaluation and online learning. This eligibility condition does not independently verify the physical accuracy of an individual radar measurement.

For each eligible event, an admission variable $g_{t}\leq V_{t}$ determines whether the observation participates in parameter adaptation. Importantly, learning admission and sequence evolution are separated: an observation rejected for model updating still remains in the subsequent measurement history. This separation allows the learner to regulate parameter adaptation without discarding valid sensing information.

### 2.2. Adaptation and Retention Objectives

The learner is initialized from source-domain measurements and subsequently adapts to a chronological deployment stream without explicit task-boundary labels or model resets. Current-stream prediction accuracy and preservation of source-domain performance are treated as complementary objectives.

Let $D_{\mathrm{source},\mathrm{test}}$ denote a held-out source set that is excluded from model fitting and hyperparameter selection. The signed retention change after event *t* is

(4) $$\Delta_{\mathrm{source}}\left(t\right)=\mathrm{MAE}\left(\theta_{t};D_{\mathrm{source},\mathrm{test}}\right)-\mathrm{MAE}\left(\theta_{0};D_{\mathrm{source},\mathrm{test}}\right).$$

A negative value indicates that the adapted model retains or improves predictive performance on the source distribution, whereas a positive value indicates degradation.

To distinguish prediction evaluation from controlled learning updates, the radar-reported range $y_{t}$ is used as the scoring target, while $y_{t}^{\mathrm{upd}}$ denotes the value supplied to the learning process:

(5) $$y_{t}^{\mathrm{upd}}=y_{t}\;\mathrm{under}\;\mathrm{natural}\;\mathrm{operation}.$$

Under controlled update-channel perturbations, only $y_{t}^{\mathrm{upd}}$ is modified. The archived prediction is evaluated against the original radar-reported range $y_{t}$, which also remains in the subsequent measurement history. Learning admission and parameter updating operate on $y_{t}^{\mathrm{upd}}$, allowing the adaptation pathway to be controlled without altering the causal prediction protocol or the original observation stream.

## 3. Joint-Gated Continual Learning Framework

**Figure 1:**
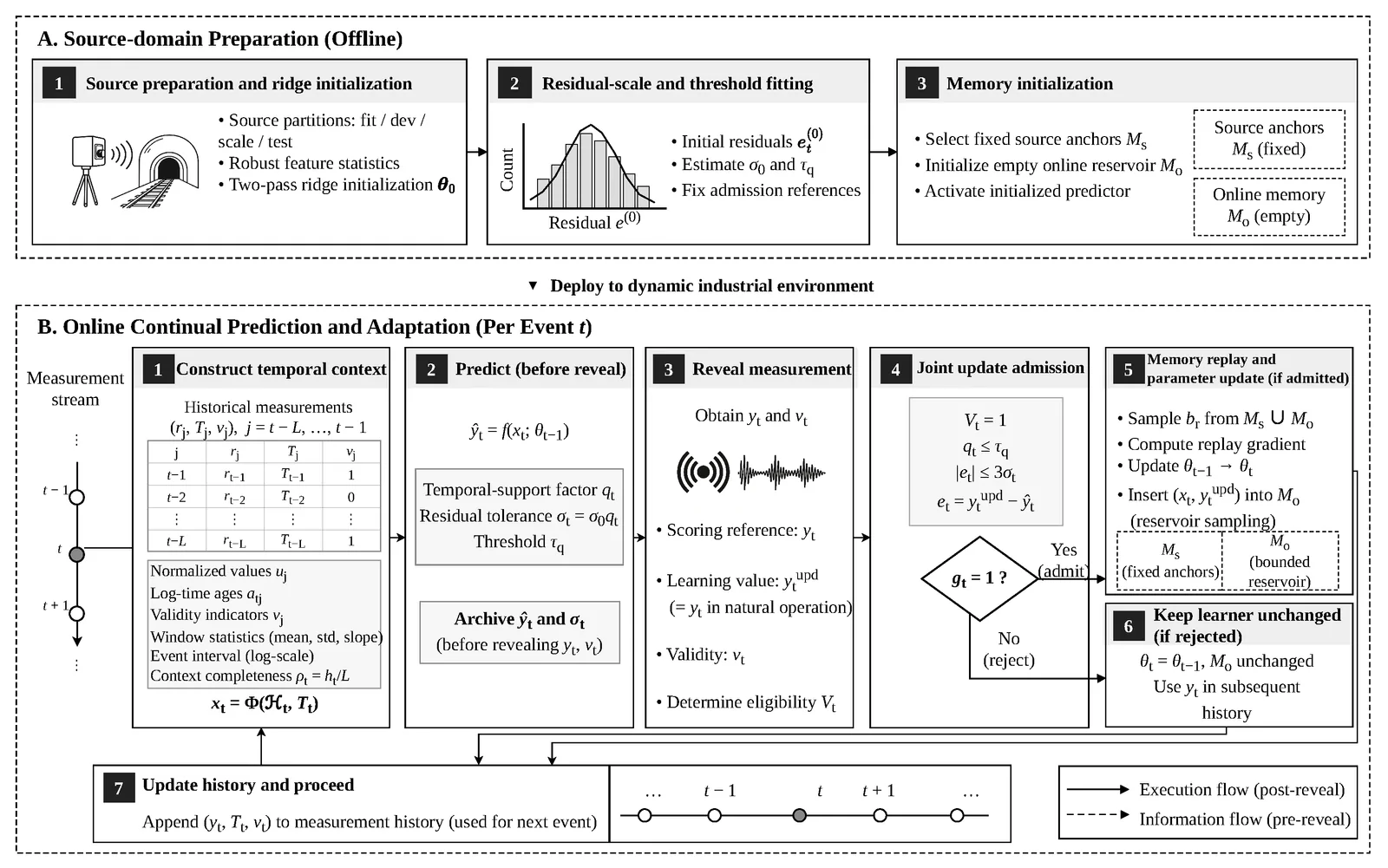
Overview of the proposed JGCL-Radar framework. Source-domain preparation determines the initial predictor, residual-scale mapping, and fixed source anchors. During deployment, each event is predicted before the current measurement is revealed. Temporal-context support and observation–prediction consistency jointly determine update admission. Admitted observations update the predictor and bounded online memory, whereas rejected observations leave the learner unchanged but remain in the observation stream for subsequent prediction. Here, “dev” denotes the source-development partition, “std” denotes standard deviation, and $e_{t}^{\left(0\right)}$ denotes the source-domain prediction residual obtained using the initialized predictor $\theta_{0}$.

Building on the causal formulation in Section 2, we develop JGCL-Radar, a joint-gated continual learning framework comprising temporal-context representation, context-support modeling, observation–prediction consistency assessment, and bounded-memory adaptation. Figure 1 separates source-domain preparation from deployment-time operation. Here, update suitability quantifies whether a revealed and valid measurement should modify the predictor under the current observable temporal context and model state. Representation scales, context-reference statistics, and admission thresholds are determined from source data and remain fixed during deployment, while only the learner parameters and bounded online memory evolve.

### 3.1. Temporal Context Representation and Predictor Initialization

The temporal representation preserves measurement magnitude, event timing, and validity as distinct channels. Let *c* and s>0 denote robust source-domain location and scale estimates, respectively. A valid historical measurement is encoded as

(6) $$u_{j}=\mathrm{clip}\left(\frac{r_{j}-c}{s},-u_{\max},u_{\max}\right),$$

whereas an invalid measurement is assigned $u_{j}=0$ together with $v_{j}=0$. Here, clip(z,l,u)=min{max(z,l),u} denotes a clipping operator that bounds the normalized measurement within a predefined interval to limit the influence of extreme values on feature representation. Its temporal age relative to the current event is represented by

(7) $$a_{tj}=\frac{\log\;\left[1+\min(T_{t}-T_{j},A_{\max})\right]}{\log(1+A_{\mathrm{ref}})}.$$

Here, $A_{\max}$ is the upper bound on the historical observation age, and $A_{\mathrm{ref}}$ is the reference scale for temporal normalization. All time intervals are expressed in seconds.

The three-channel representation therefore distinguishes a numerical measurement from missing historical information while retaining nonuniform event timing.

The feature map combines the most recent K≤L channel triples with local summary statistics: the window mean, population standard deviation, the time-normalized slope between the two most recent valid measurements, the logarithmic previous-event gap, and the temporal-context completeness:

(8) $$\rho_{t}=\frac{h_{t}}{L}.$$

The slope is set to zero when fewer than two valid measurements are available. Source-domain feature means and standard deviations define a fixed standardization, followed by coordinate-wise clipping. After augmenting the feature vector with an intercept, its dimension is d=3K+6, and the predictor is

(9) $${\hat{y}}_{t}=x_{t}^{T}\theta_{t-1}.$$

Ridge regression provides the initial predictor [23]. Let $\theta ={\left[w^{⊤},b\right]}^{⊤}$, where w and *b* denote the feature weights and intercept, respectively.

An initial ridge fit is performed on $D_{\mathrm{fit}}$. The median and scaled median absolute deviation of its residuals are then used to identify the central-residual subset $D_{\mathrm{init}}\subseteq D_{\mathrm{fit}}$. The final initialization is obtained by

(10) $$\theta_{0}=\arg\min_{\theta }\left\{\sum_{t\in D_{\mathrm{init}}}{\left(y_{t}-x_{t}^{⊤}\theta \right)}^{2}+\lambda_{r}\theta^{⊤}R\theta \right\},\;R=\mathrm{diag}\left(1,\ldots ,1,0\right).$$

Here, $\lambda_{r}$ is the ridge regularization coefficient, and the final zero diagonal entry of R excludes the intercept from regularization. Initialization uses only source-domain samples, while all eligible deployment observations remain in the common prediction-evaluation protocol.

### 3.2. Temporal-Context Support Modeling

Update suitability depends not only on the current prediction residual but also on the temporal context in which the prediction is made. Let $d_{t}$ denote the median absolute increment among the most recent *K* valid measurements, $\Delta_{t}=T_{t}-T_{t-1}$ the preceding raw-event interval, and $\rho_{t}=h_{t}/L$ the temporal-context completeness. When no valid increment is available, $d_{t}$ is set to *s*. We define the temporal-context penalty as

(11) $$\begin{array}{cc} \xi_{t} & =\min\left\{\frac{d_{t}}{d_{\mathrm{ref}}},10\right\},\\ \gamma_{t} & =\min\left\{\max\left(\frac{\Delta_{t}}{\Delta_{\mathrm{ref}}}-1,0\right),4\right\},\\ q_{t} & ={\left[1+\frac{\xi_{t}^{2}}{9}+\gamma_{t}+\left(1-\rho_{t}\right)\right]}^{1/2}. \end{array}$$

Here, $d_{\mathrm{ref}}$ is the 75th percentile of $d_{t}$ over eligible source-training examples, and $\Delta_{\mathrm{ref}}$ is the 90th percentile of inter-event intervals over assigned source-training events.

The squared variation term characterizes local range fluctuations, the interval term represents deviations beyond the reference time scale, and the completeness term reflects the proportion of valid historical observations. The normalized and bounded components are combined additively, with the square-root transformation producing the context-dependent residual-scale factor $q_{t}$. All components are derived from pre-observation history, and the associated design parameters remain fixed during deployment.

An independent source-domain residual subset maps this dimensionless context penalty to a measurement-scale tolerance. For eligible scale-estimation examples, let

(12) $$e_{t}^{\left(0\right)}=y_{t}-x_{t}^{T}\theta_{0}.$$

Let $Q_{p}\left(\cdot \right)$ denote the empirical quantile operator of order *p*, and let $F_{N(0,1)}$ denote the standard-normal cumulative distribution function, with $z_{0.95}=F_{N(0,1)}^{-1}\left(0.95\right)$.

(13) $$\begin{array}{cc} \sigma_{0} & =\max\;\left\{s_{\min},\frac{Q_{0.90}\;\left(|e_{t}^{\left(0\right)}|/q_{t}\right)}{z_{0.95}}\right\},\\ \tau_{q} & =Q_{p}\left(q_{t}\right),\;p=0.90\;\mathrm{by}\;\mathrm{default}. \end{array}$$

The default temporal-support quantile is p=0.90, determined from the source-domain scale-fitting data. This mapping remains fixed throughout deployment. Within the admissible temporal-context region, $\sigma_{t}$ adapts the consistency tolerance to local measurement dynamics; when $q_{t}>\tau_{q}$, the context-support gate closes learning admission. Thus, temporal support and observation–prediction agreement are modeled as distinct but complementary conditions.

### 3.3. Joint Update-Admission Mechanism

After the current measurement is revealed, define the update-channel innovation as

(14) $$e_{t}=y_{t}^{\mathrm{upd}}-{\hat{y}}_{t},$$

where $y_{t}^{\mathrm{upd}}=y_{t}$ during natural operation. The joint admission decision is

(15) $$g_{t}=V_{t}1\left\{q_{t}\leq \tau_{q}\right\}1\{|e_{t}\left|\leq \kappa \sigma_{t}\right\}.$$

Here, κ is the residual-consistency threshold multiplier, set to 3 by default. The temporal-support quantile *p* and residual multiplier κ jointly define the learning-admission region. Their sensitivity is evaluated with the current-sample gradient-clipping bound fixed at $3\sigma_{t}$.

The first gate evaluates whether the current prediction is sufficiently supported by its temporal context, whereas the second tests whether the revealed measurement is consistent with the archived pre-update prediction at the context-conditioned residual scale. Only eligible observations satisfying both conditions are admitted for parameter adaptation and online-memory insertion.

A large natural innovation indicates strong disagreement between the revealed observation and the current predictor. Such disagreement may arise from changing operating conditions, weak temporal-context support, or predictor mismatch. The observation remains a scoring reference and subsequent temporal input, while learning admission determines whether it modifies the current model state.

For an admitted observation, the current innovation is bounded by

(16) $${\tilde{e}}_{t}=\mathrm{clip}\left(e_{t},-3\sigma_{t},3\sigma_{t}\right).$$

The clipping is redundant under the complete joint gate but remains active in matched ablation controls that remove the consistency condition. If $g_{t}=0$, the model parameters, memory contents, occupancy, and admitted-sample count remain unchanged. The original observation nevertheless enters the temporal context of subsequent events. The gate therefore regulates the adaptation pathway rather than discarding or altering the observed measurement stream.

### 3.4. Bounded-Memory Continual Adaptation

A finite memory combines $M_{s}$ fixed source anchors with an online reservoir of capacity $M_{o}$. Source anchors are sampled without replacement from eligible source-training pairs satisfying the source-domain admission rule. At each admitted deployment event, $b_{r}$ historical pairs are sampled with replacement from the currently occupied memory pool before the new observation is inserted. This order prevents the current sample from being replayed as its own historical example and allows the source/online replay composition to follow actual memory occupancy.

For a stored pair (x,y), define the normalized squared-error gradient

(17) $$G\left(\theta ;x,y\right)=\frac{(x^{T}\theta -y)x}{1+{∥x∥}_{2}^{2}}.$$

An admitted update combines the current-example contribution with the replay gradient:

(18) $$\begin{array}{cc} G_{t}^{\mathrm{new}}\; & =-\frac{{\tilde{e}}_{t}x_{t}}{1+∥x_{t}{∥}_{2}^{2}},\\ R_{t}\; & =\frac{1}{b_{r}}\sum_{k=1}^{b_{r}}G\left(\theta_{t-1};x_{t}^{\left(k\right)},y_{t}^{\left(k\right)}\right),\\ \theta_{t}\; & =\theta_{t-1}-\eta \left(G_{t}^{\mathrm{new}}+\beta R_{t}\right), \end{array}$$

where η is the online learning rate and β controls the replay contribution. If the memory is empty, $R_{t}=0$. The normalization reduces sensitivity to feature magnitude, while joint admission and innovation clipping constrain the contribution of the current observation. Fixed source anchors maintain access to earlier source relationships, whereas the bounded online reservoir retains a representative subset of admitted deployment observations, following established rehearsal principles [24].

The first $M_{o}$ admitted online observations fill the reservoir. For the *n*th admitted observation thereafter, an existing online slot is replaced with probability $M_{o}/n$, and the replaced slot is selected uniformly. Source anchors are never replaced. The reservoir therefore represents the admitted deployment sequence rather than only a recent sliding window. Both replay sampling and parameter updating precede insertion of the current observation.

For feature dimension *d*, prediction requires O(d) operations, whereas an admitted update requires $O(\left(1+b_{r}\right)d)$ operations. The total memory payload is

(19) $$O\;\left((M_{s}+M_{o})d\right),$$

With fixed feature dimension, replay batch size, and memory capacities, the per-event prediction and update complexities and the model-memory requirement remain independent of the deployment stream length. The CPU execution cost of the continual-learning framework is further quantified experimentally.

## 4. Experiments

### 4.1. Field Dataset and Experimental Protocol

**Figure 2:**
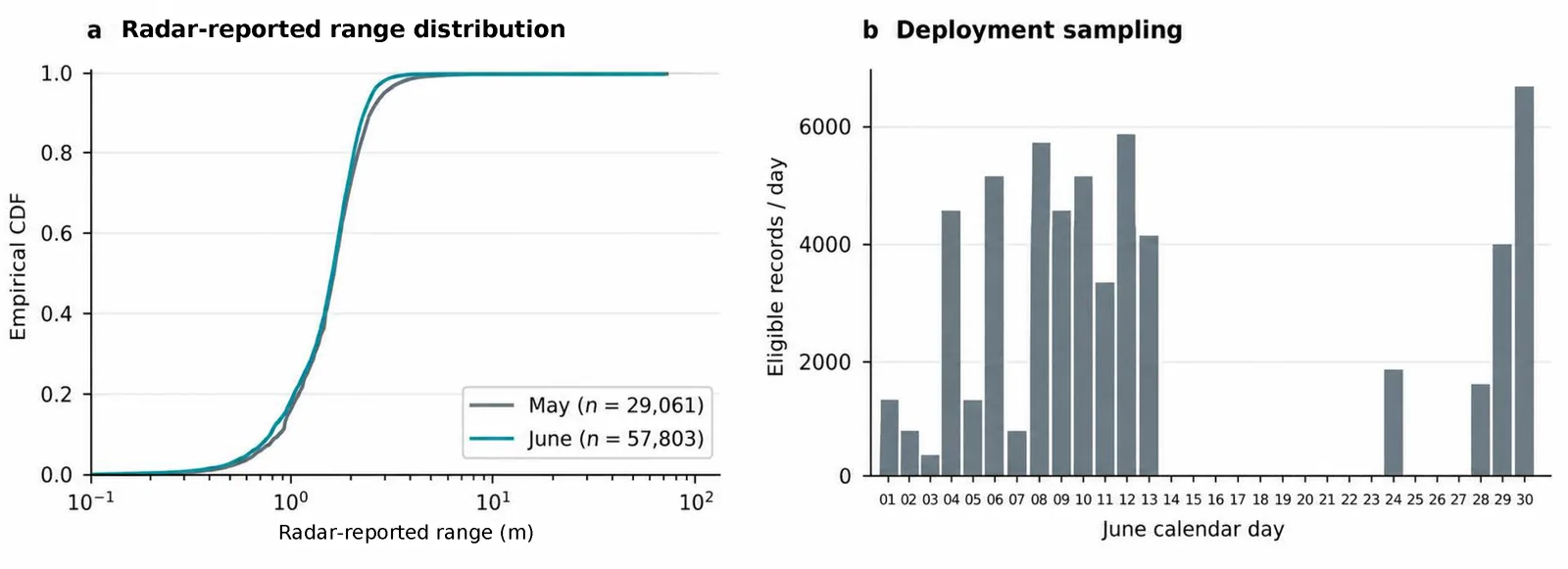
Distributional characteristics of the radar distance data and deployment sampling. (**a**) Empirical cumulative distribution functions of eligible May source-domain records and June deployment records on a logarithmic distance axis. (**b**) Daily counts of eligible deployment records over the 19 recorded days in June. All prediction experiments use the left-back (LB) radar channel.

The field measurements were collected at the excavation working face of Zhaoergu Coal Mine using roadheader-mounted GUL15 mining intrinsically safe radar sensors manufactured by Shandong Tongchuang Instrument Co., Ltd., Jining, China. The sensor employs frequency-modulated continuous-wave (FMCW) radar technology at 120 GHz, with a specified measurement range of 0.15–15 m, nominal ranging accuracy of ±3 mm, and a transmitting beam angle of $3^{{}^{\circ}}$. It supports RS485 communication using the Modbus-RTU protocol. The radar was mounted on the left-rear side of the roadheader, corresponding to the left-back (LB) channel, and oriented perpendicular to the left roadway sidewall. The recorded scalar range represents the lateral distance from the LB sensor location to the adjacent sidewall. This channel was used to evaluate continuous range prediction and learning admission under a fixed measurement direction. Before field deployment, the radar was calibrated using laser ranging and manual distance measurements. The radar-reported ranges recorded during operation served as the targets for model prediction and performance evaluation. Figure 2 summarizes the recorded-range distributions of the source and deployment data and the daily sampling pattern across the 19 recorded June dates.

The data were partitioned chronologically. May 2026 provided the source-domain fitting, development, residual-scale fitting, and held-out retention data, whereas June 2026 formed the continuous deployment stream. The May training partition contained 12,730 assigned events and 12,685 eligible targets; the validation partition contained 7956 assigned events and 7924 eligible targets; and the held-out source test contained 8481 assigned events and 8452 eligible targets. The June deployment stream contained 57,960 assigned events and 57,803 eligible targets across 19 recorded dates. Histories retained intervening raw records between assigned prediction targets and were ordered by timestamp and original row identifier.

Eligibility required a finite positive distance, valid parser status, and at least eight valid measurements within the 32-event history. Invalid historical records were encoded with zero measurement values and explicit validity indicators. All methods used the same chronological histories and common scoring masks. The chronological target assignments and source-derived parameter values are summarized in Table 1.

The fixed representation used L=32, K=8, and $h_{\min}=8$, yielding 29 standardized features and one intercept. Source-value normalization used c=median(y) and s=max(0.02,1.4826median|y−c|), with distances expressed in metres. Normalized historical values and standardized feature coordinates were clipped to [−8,8]. Temporal age encoding capped elapsed time at 86,400 s and used $A_{\mathrm{ref}}=180$ s. Feature standard deviations below $10^{-6}$ were replaced by one, and the slope denominator used a $10^{-3}$ s numerical floor. Ridge initialization used $\lambda_{r}=2$ and a central-residual cutoff of 3.5 scaled median absolute deviations with a $10^{-4}$ m scale floor.

The bounded memory contained 128 fixed source anchors and 128 online reservoir slots. Each admitted update used four pre-insertion replay samples with β=1. Seeds 13, 37, and 73 controlled sampling, replay, reservoir replacement, and random admission, while the chronological data order remained fixed. Hyperparameter selection was completed on the source fitting and development partitions, and final residual-scale fitting used the second validation half.

The comparison included Fixed, persistence, a scalar Kalman filter, recursive least squares with a forgetting factor (RLS-FF), innovation-gated recursive least squares (RLS-FF-IG), fine-tuning, elastic weight consolidation (EWC), Plain Replay, consistency-only adaptation, and four compact neural predictors. Fixed used the common ridge initialization. Persistence used the most recent valid historical measurement. The random-walk Kalman level model [25] reconstructed its level and variance within each 32-event history using a development-selected process-to-measurement variance ratio. RLS-FF performs recursive parameter estimation using eligible observations, while RLS-FF-IG further applies an innovation-consistency condition to regulate measurement admission. Both methods use the common feature representation and source-development selection protocol. The selected forgetting factor was $\lambda_{\mathrm{RLS}}=1$ for both methods. All comparisons follow the same causal prediction and evaluation protocol. Fine-tuning updated the predictor on every eligible event, whereas Plain Replay added four replay gradients. EWC [26] added $\lambda \tilde{F}⊙\left(\theta -\theta_{0}\right)$, with ${\tilde{F}}_{\ell }=E_{\mathrm{train}}\left[x_{\ell }^{2}\right]/\max_{m}E_{\mathrm{train}}\left[x_{m}^{2}\right]$. Related online parameter tracking has also been studied for classification [27]; here, the linear comparisons isolate update policies for continuous distance regression.

The long short-term memory (LSTM) network [28] and gated recurrent unit (GRU) network [29] each used one recurrent layer with 32 hidden units. The temporal convolutional network (TCN) contained two residual blocks, each with two width-32 causal convolutions, kernel size seven, and dilation one or two, giving a nominal receptive field of 37 events [30]. The Transformer [31] used two pre-normalized layers, model width 32, four attention heads, feed-forward width 128, and sinusoidal positional encoding. Neural predictors were trained using Huber loss, Adam with a learning rate of $10^{-3}$, a batch size of 256, and at most, 40 epochs with development early stopping of patience six. Their deployment parameters were fixed after source training.

### 4.2. Development-Selected Policies and Main Performance

Fine-tuning, EWC, Plain Replay, consistency-only, and JGCL-Radar were selected using mean prequential MAE across the three seeds on the source-development data. The common learning-rate grid was

$$\eta \in \{10^{-4},3\times 10^{-4},10^{-3},3\times 10^{-3},10^{-2},5\times 10^{-2},0.2\}.$$

When the optimum occurred at the lower boundary, the additional values $\left\{10^{-5},3\times \;10^{-5}\right\}$ were evaluated. EWC additionally searched λ∈{0.01,0.1,1}. The 59 candidate configurations and 177 seed runs selected $10^{-4}$ for fine-tuning and EWC, $3\times 10^{-5}$ for Plain Replay, 0.05 for consistency-only, and 0.003 for JGCL-Radar; EWC selected λ=0.01. These settings were fixed for all subsequent deployment evaluations.

Let E denote the common eligible event set, N=|E|, *S* the number of algorithm seeds, and $e_{t,s}=y_{t}-{\hat{y}}_{t,s}$. The reported point-error metrics are

(20) $$\mathrm{MAE}=\frac{1}{SN}\sum_{s=1}^{S}\sum_{t\in E}\left|e_{t,s}\right|,\;\mathrm{RMSE}=\sqrt{\frac{1}{SN}\sum_{s=1}^{S}\sum_{t\in E}e_{t,s}^{2}}.$$

Sample standard deviations are computed across seed-specific metrics. Paired 95% confidence intervals use 2000 shared whole-day cluster bootstrap resamples with identical date multiplicities across compared methods. Event-weighted MAE, squared-error means, and admission rates are recomputed in every resample. Deployment comparisons use the 19 June dates, whereas source-retention comparisons use the four held-out May dates. The prediction performance of all baseline methods and the paired policy comparisons are presented in Table 2.

On the 57,803 common eligible deployment targets, JGCL-Radar achieved an MAE of 51.07 mm and an RMSE of 206.76 mm. Relative to Fixed, MAE decreased by 0.70 mm with a paired 95% confidence interval of [−0.96,−0.43] mm, while the RMSE difference was +2.14 mm with an interval of [1.29,3.33] mm. Compared with the independently development-selected consistency-only policy, JGCL-Radar reduced RMSE from 212.17 to 206.76 mm, corresponding to a 2.55% reduction. The respective selected learning rates were 0.05 and 0.003. Matched-rate mechanism comparisons in Section 4.3 further separate parameter selection from learning admission. Kalman and persistence prediction achieved lower MAE, while fine-tuning, EWC, and Plain Replay achieved lower RMSE. The frozen GRU and Transformer predictors produced lower point estimates for both metrics. The final JGCL-Radar source MAE was 43.153 mm, corresponding to a change of −0.345 mm with a paired 95% confidence interval of [−1.079,0.078] mm relative to initialization.

### 4.3. Matched Mechanisms and Update-Channel Disturbances

The mechanism study fixed η=0.003, the selected JGCL-Radar learning rate, together with its initialization, temporal representation, and mixed-memory configuration. M1 denotes the complete joint-admission policy. M2 removes the temporal-context support condition while retaining the same context-conditioned residual scale $\sigma_{t}$. M3 applies source-defined random thinning after the consistency test. M4 retains joint admission and identical memory sampling/insertion operations while setting the replay-gradient weight to β=0.

Source residual-scale fitting produced 3375 joint admissions and 3759 consistency admissions among 3962 eligible events. M3 therefore used the fixed thinning probability

$$p_{\mathrm{thin}}=\frac{3375}{3759}.$$

Each condition started from the same initial predictor and seed-specific source anchors.

The update-channel disturbance experiment divided the deployment stream into three equal temporal blocks with injection probabilities 0.05, 0.10, and 0.20. At selected events,

(21) $$y_{t}^{\mathrm{upd}}=\left\{\begin{array}{cc} \max(0.001,y_{t}+\epsilon_{t}k\sigma_{0}), & c_{t}=1,\\ y_{t}, & c_{t}=0, \end{array}\right.\;\epsilon_{t}\in \left\{-1,1\right\}.$$

The main experiment evaluates k∈{0,3,8} across the four mechanisms and three seeds, providing 36 mechanism–amplitude–seed combinations. The matched-mechanism results, including prediction errors, source-domain changes, and update-admission rates, are summarized in Table 3.

At the matched learning rate η=0.003, the complete joint-admission policy M1 reduced RMSE relative to consistency-only admission M2 by 0.625 mm, with a paired 95% confidence interval of [−0.946,−0.390] mm. The corresponding MAE difference was 0.03 mm. M1 and random-thinning control M3 admitted 85.51% and 85.24% of eligible events, respectively. Under these nearly matched update frequencies, M1 achieved 0.278 mm lower RMSE, with a paired 95% confidence interval of [−0.437,−0.146] mm. The MAE difference was 0.02 mm, with a confidence interval including zero. Removing the replay gradient in M4 yielded 50.85 mm MAE and 205.89 mm RMSE. The effects of replay composition are further examined in Section 4.4.

At k=3, M1 admitted 49.73% of injected events and reached 52.75 mm MAE. At k=8, injected admission fell to 0.89%, whereas admission of non-injected events remained approximately 85.65%. The gate therefore increasingly suppresses update-channel deviations as their amplitude grows.

To further compare joint learning admission with classical recursive estimation, RLS-FF and RLS-FF-IG were evaluated under natural measurements and controlled update-channel perturbations. Both methods used the source-development-selected forgetting factor $\lambda_{\mathrm{RLS}}=1$. Under natural measurements, RLS-FF obtained 59.043 mm MAE and 203.497 mm RMSE, whereas innovation-gated RLS-FF-IG achieved 50.421 mm MAE and 206.908 mm RMSE. At perturbation amplitudes k=3 and k=8, RLS-FF-IG obtained MAE values of 50.517 mm and 50.465 mm, respectively. The paired MAE differences between JGCL-Radar and RLS-FF-IG were +0.653[0.490,0.808] mm at k=0, +2.228[1.434,3.037] mm at k=3, and +0.709[0.549,0.835] mm at k=8. The corresponding RMSE confidence intervals included zero. These comparisons quantify the predictive behavior of classical innovation-gated recursive estimation under the same chronological evaluation protocol. The detailed performance of the two classical RLS baselines under different perturbation amplitudes is reported in Table 4.

**Figure 3:**
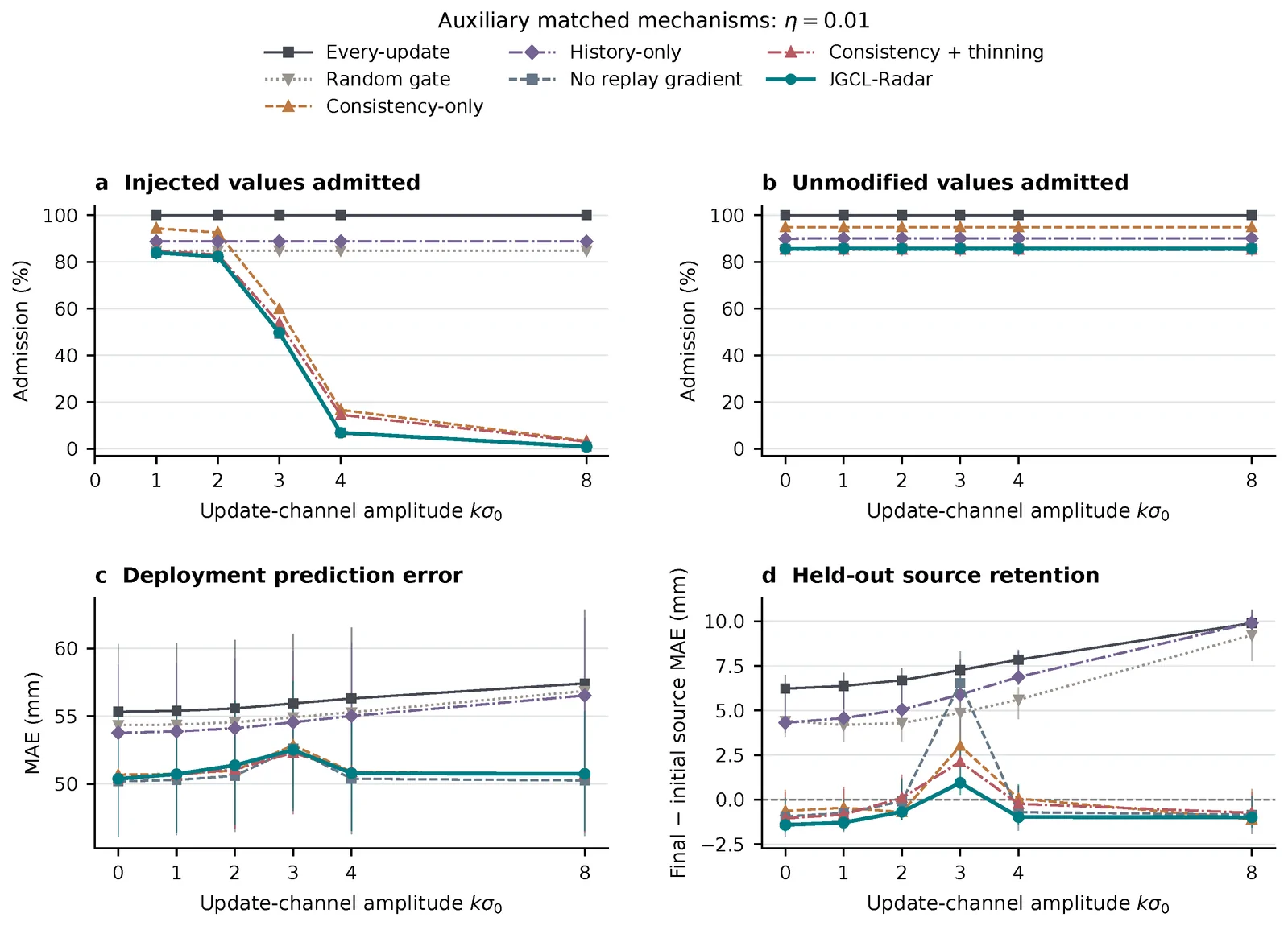
Full auxiliary amplitude scan at η=0.01. (**a**,**b**) Injected and non-injected admission rates; (**c**) deployment MAE; (**d**) final signed source-MAE change for all seven variants. Injection positions and signs are shared within each seed. Whiskers denote paired-day bootstrap intervals.

An auxiliary experiment at η=0.01 evaluates all seven admission variants over k∈{0,1,2,3,4,8}. In addition to M1–M4, Every-update admits all eligible events, Random gate uses probability 3375/3962, and History-only retains the temporal-context condition without the prediction-consistency test. At k=0, joint admission achieved 50.379 mm MAE, compared with 50.683 mm for consistency-only and 50.419 mm for thinning. At k=3, joint-admission MAE increased to 52.511 mm, and the signed source change reached +0.940 mm. The complete amplitude response is shown in Figure 3.

The sensitivity of joint admission to its threshold parameters was evaluated using p∈{0.85,0.90,0.95} for temporal-support quantiles and κ∈{2,3,4} for residual-consistency thresholds. The learning rate remained fixed at η=0.003, together with the feature representation, source-fitted residual scale, and memory configuration. The current-sample gradient-clipping bound was maintained at $3\sigma_{t}$. The nine configurations were evaluated under natural measurements and update-channel perturbations at k=3 and k=8. Under natural measurements, MAE ranged from 50.988 to 51.394 mm across the threshold combinations. The corresponding ranges were 51.072–52.745 mm at k=3 and 51.026–51.476 mm at k=8. The original configuration, p=0.90 and κ=3, yielded 51.074, 52.745, and 51.174 mm MAE under the three conditions, respectively. These results characterize the joint influence of temporal-support and residual-consistency thresholds on learning admission, with greater sensitivity under intermediate-amplitude update-channel perturbations. The complete gate-threshold sensitivity results under natural measurements and controlled perturbations are presented in Table 5.

### 4.4. Replay Composition, Sustained Changes, and Error Attribution

The auxiliary replay-composition study fixed the joint gate, η=0.01, and a total memory capacity of 256. D0 retained mixed memory with zero replay-gradient weight; D1 used 256 fixed source anchors; D2 used a 256-slot online reservoir; and D3 used 128 source anchors together with 128 online slots. Each occupied memory pool supplied four replay samples before insertion of the current admitted event.

At η=0.01, mixed storage reduced deployment MAE relative to source-only and online-only by 0.19[0.08,0.28] mm and 0.21[0.04,0.35] mm, respectively. Its differences from D0 were +0.19[−0.06,0.45] mm in deployment MAE and −0.47[−1.13,1.01] mm in source MAE.

At the final learning rate, M1 source MAE exceeded M2 by 0.771[0.109,1.103] mm, whereas the M1–M3 and M1–M4 confidence intervals crossed zero. The per-date analysis shows source-MAE changes of −0.17, −1.26, +4.25, and −0.09 mm for M1 across the four source dates. Leave-one-date-out evaluations retained a positive M1–M2 difference of 0.213–0.911 mm, while the M1–M3 difference ranged from −0.058 to 0.388 mm. The replay-composition results and source-domain stability across evaluation dates are summarized in Table 6.

Four controlled sustained-change scenarios were constructed to examine online adaptation under evolving operating conditions. At approximately one-third of the deployment stream, step changes of $+2\sigma_{0}$ and $+4\sigma_{0}$ were introduced, together with ramp changes reaching the same amplitudes over 512 raw events. The modified valid measurements were used consistently for prediction scoring, online updating, and subsequent temporal inputs, while source-derived normalization, residual scales, and admission thresholds remained fixed. Local performance was evaluated using common non-overlapping 128-event windows over the same pre- and post-change event range.

**Figure 4:**
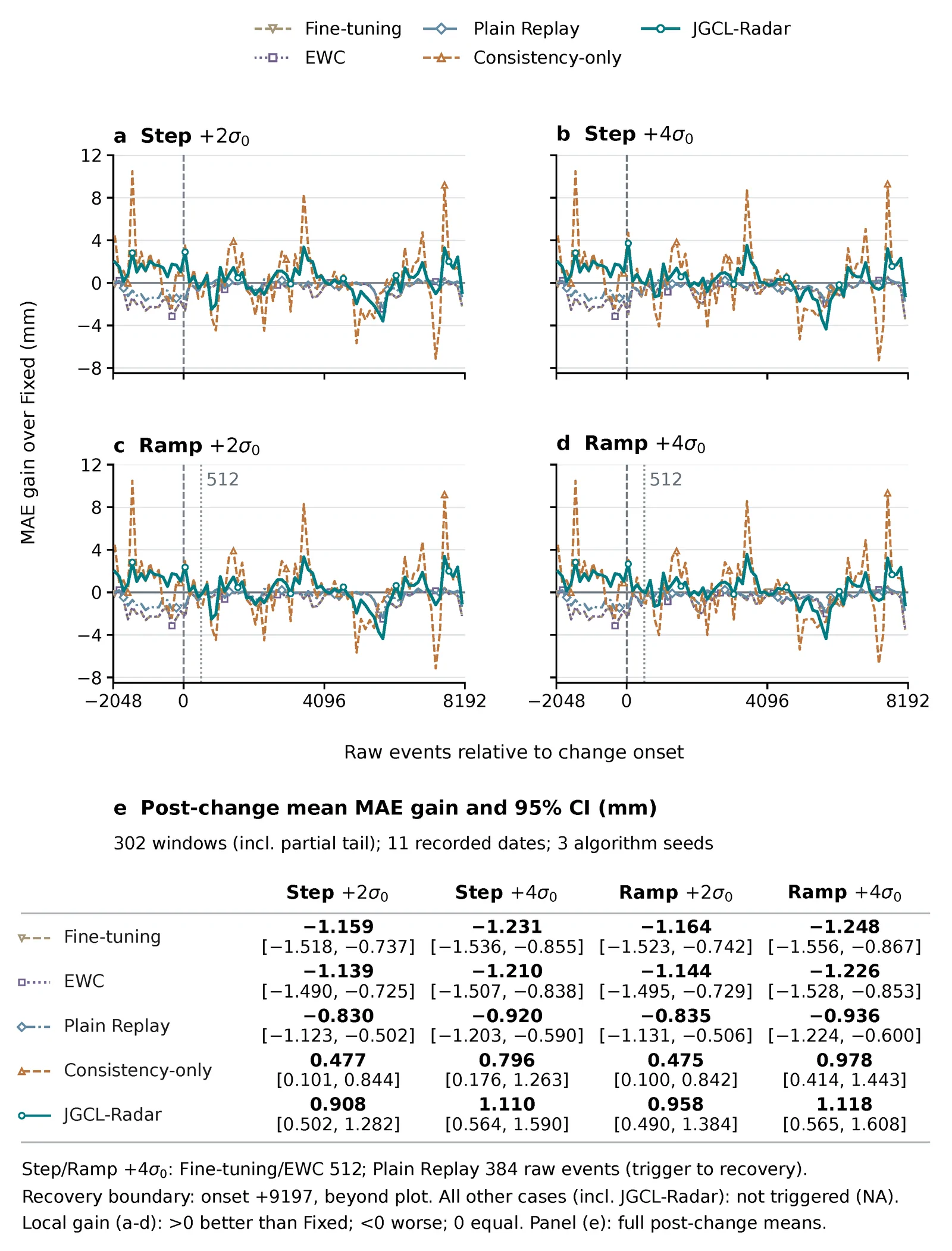
Local adaptation effects under four sustained observation-process changes. Panels (**a**–**d**) correspond to Step $+2\sigma_{0}$, Step $+4\sigma_{0}$, Ramp $+2\sigma_{0}$, and Ramp $+4\sigma_{0}$, respectively. Each panel reports the MAE gain of the adaptive policies relative to the Fixed predictor in common non-overlapping 128-event windows, defined as ${\mathrm{MAE}}_{\mathrm{Fixed}}-{\mathrm{MAE}}_{\mathrm{policy}}$; positive values therefore indicate lower prediction error than Fixed. Dashed vertical lines mark the change onset, while the dotted lines and “512” labels in the ramp scenarios mark completion of the gradual transition. Curves aggregate the three algorithm seeds. Panel (**e**) reports the post-change mean MAE gains and corresponding 95% confidence intervals for all five adaptive policies.

Figure 4 characterizes the local predictive gains of adaptive policies relative to Fixed under four sustained observation changes. For each common 128-event window, the gain is defined as ${\mathrm{MAE}}_{\mathrm{Fixed}}-{\mathrm{MAE}}_{\mathrm{policy}}$, with positive values indicating lower prediction error for the adaptive policy. The quantitative analysis covered 11 recorded dates and 302 windows (including partial tail). JGCL-Radar achieved mean post-change gains of 0.908, 1.110, 0.958, and 1.118 mm under Step $+2\sigma_{0}$, Step $+4\sigma_{0}$, Ramp $+2\sigma_{0}$, and Ramp $+4\sigma_{0}$, respectively. The corresponding paired 95% confidence intervals were [0.502,1.282], [0.564,1.590], [0.490,1.384], and [0.565,1.608] mm. Recovery diagnostics for all adaptive policies are presented in Figure 4. These results quantify local prediction gains during sustained observation changes under the evaluated adaptation policies. The mean post-change MAE gains and corresponding 95% confidence intervals of JGCL-Radar across the four scenarios are summarized in Table 7.

Tail-error attribution uses natural η=0.003 predictions from M1–M4 and Fixed. For each event, define $\Delta {\mathrm{SSE}}_{t}$ as the seed-averaged squared error of a mechanism minus that of Fixed. A common descending ranking by Fixed absolute error defines the retrospective top-1% and top-5% subsets containing 579 and 2891 events, respectively. Temporal-context support strata are defined using the frozen source-training thresholds.

JGCL-Radar added 50.839 m^2^ squared error relative to Fixed. The top 1% contributed 42.102 m^2^, corresponding to 82.81% of the net increase. The top 5% contributed 58.025 m^2^, while the remaining 95% reduced squared error by 7.186 m^2^. The median seed-averaged absolute error was 8.981 mm for JGCL-Radar and 9.472 mm for Fixed, whereas the 99th percentiles were 745.249 mm and 706.507 mm, respectively.

**Figure 5:**
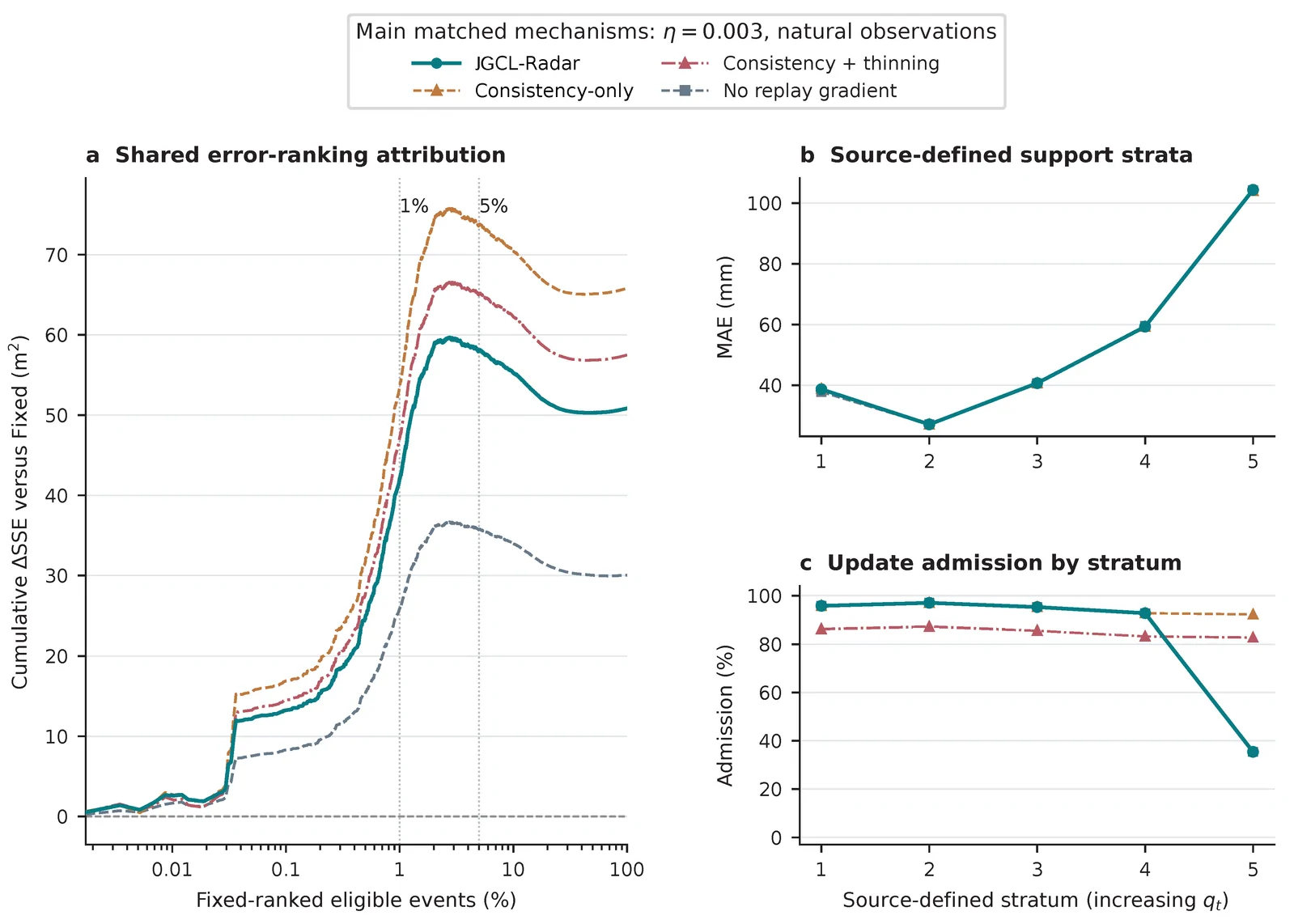
Main-configuration error attribution at η=0.003. (**a**) Cumulative squared-error differences from Fixed over all 57,803 eligible events ranked by descending Fixed absolute error; dotted guides mark the top 1% and 5%. (**b**,**c**) MAE and update admission across five source-defined temporal-context support strata containing 13,611, 12,822, 11,393, 10,473, and 9504 deployment events. Positive ΔSSE indicates greater squared error than Fixed.

Within the weakest source-defined temporal-context stratum, containing 9504 deployment events, JGCL-Radar obtained 104.409 mm MAE with a 35.46% admission rate. Figure 5 links the concentration of tail error to progressively weaker temporal-context support.

### 4.5. Computational Efficiency

Experiments were conducted on a Linux server equipped with an Intel Xeon Platinum 8336C central processing unit (CPU) at 2.30 GHz and an NVIDIA RTX 4090 graphics processing unit (GPU). Deployment-time CPU measurements were performed under a common single-thread protocol with batch size one, preloaded models, and ten repeated runs. Computational costs were measured as per-event prediction latency and complete deployment-stream processing time over 57,960 assigned events.

JGCL-Radar required an average of 1.060 μs per prediction and 2.676±0.008 s to process the deployment stream. For the frozen GRU predictor, the corresponding measurements were 546.617 μs per prediction and 31.846±0.086 s per stream. The admission and update component of JGCL-Radar averaged 44.505 μs per event, while the accepted-event processing path averaged 51.783 μs per event. The predictor contains 30 float64 coefficients occupying 240 bytes. The total persistent-state array footprint, including model coefficients, is 87,280 bytes. The same-CPU prediction latency and complete-stream processing times of all ten predictors are compared in Table 8.

Thirty float64 model coefficients require 240 bytes. The 256 stored feature–target pairs require 256×31×8=63,488 bytes. Identifiers and stage labels add 23,552 bytes, giving 87,040 bytes of memory arrays. The resulting storage requirement is bounded by $O(\left(M_{s}+M_{o}\right)d)$ and remains independent of deployment-stream length.

## 5. Discussion

The central mechanism of JGCL-Radar is the separation between continuous measurement utilization and selective parameter adaptation. Temporal-context support is characterized by recent range variation, event intervals, and measurement validity to establish a state-conditioned residual tolerance. Combined with the consistency between the newly revealed observation and prior prediction, this temporal support jointly regulates parameter updates and online-memory insertion. Measurements not admitted for learning remain available in subsequent histories, decoupling continuous information utilization from dynamic model adaptation. This establishes a continual prediction mechanism integrating temporal-state representation, joint learning admission, and bounded parameter adaptation.

Matched experiments further characterize the contribution of joint learning admission. At the common learning rate η=0.003, JGCL-Radar reduced RMSE by 0.625 mm and 0.278 mm relative to consistency-only admission and approximately rate-matched random thinning, respectively, indicating that temporal-support-aware selection can improve the prediction-error distribution under controlled update frequencies. Relative to independently development-selected consistency-only adaptation, RMSE decreased by 2.55%, reflecting the combined effects of learning admission and its selected parameter configuration. Compared with the fixed predictor, MAE decreased by 0.70 mm while RMSE increased by 2.14 mm; classical innovation-gated recursive least squares achieved an MAE of 50.421 mm. These results characterize the condition-dependent predictive benefits of jointly modeling temporal support and observation–prediction consistency.

Replay and controlled-disturbance experiments further reveal the dynamic adaptation characteristics of the framework. Removing the replay gradient reduced natural-stream MAE and RMSE, while mixed memory achieved lower deployment MAE than source-only and online-only memory at the auxiliary learning rate, illustrating how memory composition influences parameter evolution. Under the larger perturbation amplitude k=8, joint gating rejected 99.11% of injected learning values, whereas intermediate-amplitude disturbances exhibited greater sensitivity to admission thresholds. Across four sustained observation-change scenarios, the mean post-change MAE gain relative to Fixed ranged from 0.908 to 1.118 mm. Tail-error attribution further showed that the top 1% of high-error events accounted for 82.81% of the net squared-error increase relative to Fixed, characterizing the relationship among learning admission, local adaptation, and prediction-error distributions.

Computational evaluation further demonstrates the practical value of the proposed method. Under the same single-thread CPU protocol with batch size one, JGCL-Radar required 1.060 μs per prediction and 2.676 s to process the complete deployment stream, compared with 546.617 μs and 31.846 s for the frozen GRU predictor, respectively. The GRU achieved lower prediction errors, while JGCL-Radar combined a lightweight regression predictor and bounded memory with low-cost selective online adaptation, illustrating the trade-off between predictive performance and computational resources. These findings provide a practical approach to continuous radar range prediction and online model maintenance in underground excavation. Future work will extend the evaluation to multiple radar channels, longer operating periods, and diverse working conditions to investigate cross-scenario adaptation.

## 6. Conclusions

This paper proposes JGCL-Radar, a joint-gated continual learning method for continuous range prediction using roadheader-mounted millimeter-wave radar in coal-mine excavation. The method integrates pre-observation temporal-context support with post-observation prediction consistency to establish a joint learning-admission mechanism, selectively regulating parameter updates and online-memory insertion according to the historical measurement state and the agreement between newly revealed observations and prior predictions. By decoupling observation-stream evolution from model adaptation, measurements rejected for learning remain available for subsequent temporal modeling. Fixed source anchors and bounded online memory further enable continual model adaptation with finite storage requirements.

Experiments on 57,803 eligible field radar records showed that JGCL-Radar achieved a mean absolute error (MAE) of 51.07 mm, representing a 1.35% reduction relative to the fixed predictor, while its root mean squared error (RMSE) was 206.76 mm compared with 204.63 mm for Fixed. Relative to the independently development-selected consistency-only policy, RMSE decreased by 2.55%, and the update admission rate decreased from 94.83% to 85.51%. At the matched learning rate η=0.003, joint admission reduced RMSE by 0.625 mm and 0.278 mm relative to consistency-only admission and approximately rate-matched random thinning, respectively, demonstrating the contribution of jointly modeling temporal-context support and prediction consistency to learning-sample selection. Classical innovation-gated recursive least squares achieved an MAE of 50.421 mm, while the frozen GRU and Transformer obtained lower values of both error metrics, reflecting the distinct predictive characteristics of different model architectures.

Controlled disturbance experiments further showed that joint gating rejected 99.11% of injected learning values under large-amplitude update-channel perturbations, while intermediate-amplitude disturbances exhibited sensitivity to admission thresholds. In the same-CPU evaluation, JGCL-Radar achieved an average prediction latency of 1.060 μs and processed the complete deployment stream in 2.676 s, demonstrating the computational efficiency of lightweight regression and bounded-memory continual learning. Overall, JGCL-Radar integrates temporal-state awareness, joint learning admission, and the decoupling of measurement utilization from model adaptation to enable computationally efficient selective continual learning. The framework provides a technical basis for continuous sidewall-range prediction and online model maintenance in dust- and mist-affected excavation environments, with potential integration into multidirectional clearance sensing for assisted autonomous roadheader guidance. Future work will extend the evaluation to multiple radar channels, longer operating periods, and diverse working conditions to investigate cross-scenario adaptation. 

## Figures and Tables

**Table 1:** Chronological target assignments and source-derived algorithm values.

**A. Data partitions**				
Partition	Assigned	Eligible	Start date	End date
May training	12,730	12,685	11 May 2026	20 May 2026
May validation	7956	7924	20 May 2026	28 May 2026
May held-out test	8481	8452	28 May 2026	31 May 2026
June deployment	57,960	57,803	1 June 2026	30 June 2026
**B. Source-derived values and numerical floors**				
Quantity	Value	Derivation		Floor
$d_{\mathrm{ref}}$	0.024 m	Training eligible volatility, $Q_{0.75}$		0.001 m
$\Delta_{\mathrm{ref}}$	7 s	Training assigned gap, $Q_{0.90}$		1 s
$\sigma_{0}$	0.0598 m	Source-residual scale fitting		0.02 m
$\sigma_{0}\tau_{q}$	0.0839 m	Fitted scale × $Q_{0.90}\left(q_{t}\right)$		—

Assigned events include both eligible and ineligible targets. Each validation half contains 3978 assigned events and 3962 eligible targets. Contiguous partitions may share calendar dates while retaining disjoint assigned row identifiers. The volatility reference is estimated from eligible training histories, whereas the event-gap reference is estimated from assigned training events. All source-derived values are fixed before deployment.

**Table 2:** Development-selected policies and retained predictors on the common deployment targets. Errors and signed source changes are in mm.

**A. Main performance and algorithm-seed variation**				
Predictor	MAE ± SD	Pooled RMSE ± SD	$\Delta_{s}\;\pm$ SD	Update (%)
Fixed	51.77±0.00	204.63±0.00	0.00±0.00	0.00
Fine-tuning	52.47±0.00	204.49±0.00	1.58±0.00	100.00
EWC	52.46±0.00	204.49±0.00	1.55±0.00	100.00
Plain Replay	52.24±0.48	204.57±0.05	0.99±1.18	100.00
Consistency-only	50.99±0.46	212.17±0.35	−0.40±0.67	94.83
JGCL-Radar	51.07±0.39	206.76±0.40	−0.34±0.61	85.51
Persistence	49.83±0.00	265.94±0.00	0.00±0.00	0.00
Kalman	48.40±0.00	216.63±0.00	0.00±0.00	0.00
LSTM	51.71±1.48	199.12±0.90	0.00±0.00	0.00
GRU	49.45±0.79	198.20±0.17	0.00±0.00	0.00
TCN	51.56±0.97	199.99±0.40	0.00±0.00	0.00
Transformer	49.34±1.80	199.73±0.97	0.00±0.00	0.00
**B. Paired policy differences: JGCL-Radar minus comparator**				
Comparator	MAE difference [95% CI]		RMSE difference [95% CI]	
Fixed	−0.70[−0.96,−0.43]		2.14[1.29,3.33]	
Consistency-only (η=0.05)	0.09[−0.31,0.52]		−5.41[−6.32,−4.55]	

All methods evaluate the same 57,803 eligible deployment targets. SD denotes variation across the three algorithm seeds. Negative $\Delta_{s}$ indicates lower held-out source MAE after deployment than at initialization. Panel B uses 2000 paired whole-day cluster bootstrap resamples and each method’s development-selected learning rate.

**Table 3:** Matched mechanisms at the final learning rate η=0.003 under natural measurements and update-channel disturbances.

**A. Absolute performance and admission**					
*k*	Mechanism	MAE ± SD	Pooled RMSE ± SD	$\Delta_{s}\;\pm$ SD	Admission (%) Total/Injected/Other
0	M1	51.07±0.39	206.76±0.40	−0.34±0.61	85.51/–/85.51
	M2	51.05±0.08	207.39±0.16	−1.12±0.48	94.87/–/94.87
	M3	51.05±0.17	207.04±0.55	−0.63±0.24	85.24/–/85.24
	M4	50.85±0.00	205.89±0.00	−0.46±0.00	85.48/–/85.48
3	M1	52.75±0.39	206.77±0.13	2.71±0.56	81.48/49.73/85.65
	M2	52.39±0.26	207.20±0.43	1.83±1.05	90.73/59.45/94.85
	M3	52.66±0.36	206.78±0.55	2.08±1.48	81.54/53.26/85.26
	M4	52.82±0.12	205.65±0.04	3.48±0.67	81.42/49.36/85.64
8	M1	51.17±0.11	206.71±0.28	−0.67±0.14	75.80/0.89/85.65
	M2	50.92±0.14	207.46±0.47	−1.26±0.37	84.21/3.29/94.85
	M3	51.38±0.12	206.99±0.32	−0.56±0.22	75.67/3.08/85.22
	M4	50.89±0.01	205.81±0.02	−0.50±0.04	75.78/0.90/85.63
**B. Paired differences: M1 minus matched comparator**					
*k*	Comparator	ΔMAE [95% CI]	ΔRMSE [95% CI]	ΔSource MAE [95% CI]	
0	M2	0.03[−0.08,0.12]	−0.63[−0.95,−0.39]	0.77[0.11,1.10]	
	M3	0.02[−0.09,0.12]	−0.28[−0.44,−0.15]	0.29[−0.23,0.48]	
	M4	0.22[0.09,0.34]	0.87[0.51,1.39]	0.12[−0.0013,0.66]	
3	M2	0.36[0.15,0.56]	−0.43[−0.68,−0.23]	0.88[0.66,1.05]	
	M3	0.09[−0.14,0.36]	−0.0048[−0.11,0.08]	0.62[0.28,1.81]	
	M4	−0.07[−0.51,0.23]	1.12[0.72,1.70]	−0.77[−1.00,0.66]	
8	M2	0.25[0.14,0.34]	−0.75[−1.25,−0.36]	0.59[−0.16,0.88]	
	M3	−0.21[−0.29,−0.12]	−0.28[−0.59,−0.05]	−0.11[−0.37,−0.0004]	
	M4	0.28[0.18,0.38]	0.90[0.61,1.31]	−0.17[−0.32,0.32]	

All error quantities and paired differences are in mm. M1: joint admission with mixed replay; M2: consistency-only admission; M3: consistency admission with source-defined thinning; M4: joint admission with zero replay-gradient weight and identical memory operations. Final source MAE equals the common initial value of 43.4978 mm plus $\Delta_{s}$.

**Table 4:** Classical recursive least-squares baselines under natural measurements and controlled update-channel perturbations. All error quantities are in mm.

Method	*k*	MAE	RMSE	$\Delta_{s}$
RLS-FF	0	59.043	203.497	6.358
RLS-FF-IG	0	50.421	206.908	−1.772
RLS-FF	3	59.054	203.502	6.337
RLS-FF-IG	3	50.517	206.677	−1.638
RLS-FF	8	59.111	203.515	6.347
RLS-FF-IG	8	50.465	206.684	−1.712

**Table 5:** Gate-threshold sensitivity under natural measurements and update-channel perturbations. Entries report deployment MAE in mm at the fixed learning rate η=0.003.

*p*	κ	k=0	k=3	k=8
0.85	2	51.239	51.308	51.412
0.85	3	51.188	52.632	51.139
0.85	4	51.292	51.552	51.476
0.90	2	51.198	51.134	51.223
0.90	3	51.074	52.745	51.174
0.90	4	51.394	51.940	51.280
0.95	2	51.034	51.072	51.114
0.95	3	50.988	52.738	51.026
0.95	4	51.045	51.625	51.173

**Table 6:** Replay composition at the auxiliary learning rate and source-date stability at the final learning rate.

**A. Auxiliary memory comparison: η=0.01, natural measurements**					
Condition		MAE ± SD	Pooled RMSE ± SD	Source MAE ± SD	$\Delta_{s}\;\pm$ SD
D0: No-replay-gradient		50.19±0.00	208.06±0.00	42.55±0.00	−0.95±0.00
D1: Source-only		50.57±0.30	209.87±1.26	42.20±0.33	−1.30±0.33
D2: Online-only		50.59±0.48	209.92±0.71	42.51±0.51	−0.99±0.51
D3: Mixed		50.38±0.42	209.85±1.38	42.08±0.48	−1.42±0.48
**B. Final-rate source stability: η=0.003, k=0**					
Source-test date	*n*	M1 Δ± SD	M2 Δ± SD	M3 Δ± SD	M4 Δ± SD
28 May 2026	5257	−0.17±0.90	−1.28±0.66	−0.67±0.11	−0.17±0.00
29 May 2026	1686	−1.26±0.34	−1.57±0.14	−1.36±0.56	−1.27±0.00
30 May 2026	58	4.25±1.57	3.87±2.33	5.05±0.70	1.75±0.00
31 May 2026	1451	−0.09±0.31	−0.19±0.34	0.12±0.69	−0.68±0.00

All error quantities are in mm. Panel A compares four memory compositions at the auxiliary learning rate. Panel B evaluates the four matched mechanisms from Table 3 on the four held-out source dates using frozen adapted parameter states.

**Table 7:** Post-change predictive gains of JGCL-Radar relative to Fixed under sustained observation changes.

Scenario	Mean Gain (mm)	95% CI (mm)
Step $+2\sigma_{0}$	0.908	[0.502, 1.282]
Step $+4\sigma_{0}$	1.110	[0.564, 1.590]
Ramp $+2\sigma_{0}$	0.958	[0.490, 1.384]
Ramp $+4\sigma_{0}$	1.118	[0.565, 1.608]

**Table 8:** Same-CPU computational performance of ten predictors under a single-thread, batch-size-one protocol. Results are based on ten repeated runs over 57,960 assigned deployment events.

Predictor	Prediction (μs)	Stream (s)	SD (s)
Fixed	1.128	0.171	0.027
Fine-tuning	1.122	0.420	0.010
EWC	1.055	0.536	0.002
Plain Replay	1.058	2.696	0.020
Consistency-only	1.064	2.950	0.019
JGCL-Radar	1.060	2.676	0.008
LSTM	138.805	8.261	0.029
GRU	546.617	31.846	0.086
TCN	475.246	27.783	0.108
Transformer	405.849	23.720	0.078

Prediction denotes mean per-event inference latency excluding online parameter updates. Stream denotes mean processing time for the complete deployment sequence, including each method’s deployment-time operations. SD denotes the standard deviation across ten repeated timing runs.

## Data Availability

The datasets presented in this article are not readily available to technical limitations. Requests to access the datasets should be directed to corresponding author.
